# Isolation and characterization of peroxidase P7-like gene and Rab-GDI like gene from potential medicinal plants: A step toward understanding cell defense signaling

**DOI:** 10.3389/fpls.2022.975852

**Published:** 2022-09-02

**Authors:** Raheela Jabeen, Atia Iqbal, Farah Deeba, Faisal Zulfiqar, Ghulam Mustafa, Haq Nawaz, Ume Habiba, Muhammad Nafees, Abbu Zaid, Kadambot H. M. Siddique

**Affiliations:** ^1^Department of Biochemistry and Biotechnology, The Women University Multan, Multan, Punjab, Pakistan; ^2^Department of Microbiology and Molecular Genetics, The Women University Multan, Multan, Punjab, Pakistan; ^3^Department of Horticultural Sciences, Faculty of Agriculture and Environment, The Islamia University of Bahawalpur, Bahawalpur, Pakistan; ^4^Department of Biochemsitry, Government College University Faisalabad, Faisalabad, Punjab, Pakistan; ^5^Department of Biochemistry, Bahauddin Zakariya University, Multan, Punjab, Pakistan; ^6^Aligarh Muslim University, Aligarh, Uttar Pradesh, India; ^7^University of Western Australia, Perth, WA, Australia

**Keywords:** defensin gene, *Albizia lebbeck*, *Moringa oleifera*, *in silico* analysis, plant defense system

## Abstract

Defensin genes form part of a plant’s defense system and are activated when exposed to biotic or abiotic stress. They play a vital role in controlling many signaling pathways involved in various plant defense mechanisms. This research aimed to isolate and characterize novel defensin genes from selected medicinally important plants to explore their signaling mechanisms and defense associated roles for breeding. The DNA of *Albizia lebbeck* and *Moringa oleifera* was subjected to PCR amplification using gene-specific primers of defensin genes. Two novel defensin genes were isolated in each species, with sequence lengths of 300 bp in *A. lebbeck* and 150 bp in *M. oleifera*. *In-silico* analysis undertaken to retrieve and align their orthologous sequences revealed 100% similarity of the *A. lebbeck* gene with the *Musa acuminate* peroxidase P7-like gene and 85% similarity of the *M. oleifera* gene with the *Manihot esculenta* GDP dissociation inhibitor gene. The reliability, stability and physiochemical properties of homology models of these sequences was confirmed through online computational studies. This preliminary study confirmed the presence of novel genes with peroxidase P7 and Rab GDP dissociation inhibitor gene-like activity in *A. lebbeck* and *M. oleifera*, respectively, and their potential defense role in plants. Thus, the defensin genes of both species could be used in the synthesis of transgenic self-defensive plants with increased disease resistance and as potential candidates for improved crop production and thraputic formulation in the future.

## Introduction

Plants are often exposed to biotic and abiotic stresses and use their natural defense system to circumvent them (Chojak-Koźniewska et al., 2018), producing several metabolites and secondary messengers to guard against these assaults (Reichling, 2018). Once the stress is detected, signaling pathways are triggered that induce gene expression to help plants acclimate to these conditions (Novaković et al., 2018). Among these, antimicrobial peptides (AMPs) form the first line of defense in a plant’s protective mechanisms (Mahlapuu et al., 2016) and are higly important part of innate immunity (Zharkova et al., 2019), serving an antimicrobial function (Rawat et al., 2017). Defensins belong to the predominant families of AMPs. They are small (45–55 amino acids), non-acidic, rich in cysteine residues, and highly similar to peptides in many other organisms (Al Akeel et al., 2014). Plant defensins involve mechanisms to inhibit fungi and bacteria, reportedly by the structural targets in the cell membrane of microbes by producing reactive oxygen species (Parisi et al., 2019). They can also protect the host by inducing and potentiating several other plant-defending mechanisms (Souza et al., 2017). The isolation and characterization of these defensins are vital for developing economically and medically important substances. Analysis of sequenced plant genomes revealed that defensins are present as multigene families and overrepresented in the genomes of some plant species (Kovaleva et al., 2017). Cowpea defensin Cp-thionin II was isolated and characterized, with its antifungal activity checked against *F. culmorum* (Schmidt et al., 2019). Similarly, two tomato defensin genes (*SlyDF1* and *SlyDF2*) were cloned and characterized from *Solanum lycopersicum* (Cui et al., 2018). The AMPs are not only have their importance in plant defense system, but animals have also AMPs as their part of immune system, e.g., three novel β-defensin AMPs were also characterized in rainbow trout (*Oncorhynchus mykiss*; Casadei et al., 2009).

With the wealth of defensin nucleotide sequences available, gene isolation strategies coupled with recombinant production are used increasingly to characterize closely related plant defensin peptides. Many public databases and tools are available to study the structure and physicochemical properties of plant genes and transcriptomes (Lakshmi Priya and Sabu, 2015).

*Albizzia lebbeck* and *Moringa oleifera* have therapeutic potential against several diseases. *Albizzia lebbeck* bark extract is very much effective against speck disease (Arasu et al., 2022). The aquous extract of the same plant have reported anti-inflammatory activity (Kamala Lakshmi and Valarmathi, 2020). similarly the plant also possesses antidiabetic and anti lipedemic effect (Azam et al., 2022). *Moringa oleifera* also have several medicinal properties like anti diabetic, anti cancerous and anti inflammatory etc. (Kumar, 2017). Considering the importance of these medicinal plants, this study resulted in isolation and characterizion of defensin genes from leaf samples of *Albizia lebbeck* and *Moringa oleifera*. *In silico* analysis of these isolates revealed the properties and functional similarty of these novel genes with peroxidase P7 and Rab GDP dissociation inhibitor like gene activities, respectively. Physiochemical and homology modeling characterization studies revealed high similarity index of 100% and 85% for genes from *A. lebbeck* and *M. oleifera* with reliable structural stability properties. This preliminary study provides the data which may help us to understand the defense mechanism and transgenic plant breed optimization. Future experimental study of these genes may provide a pipeline for drug discovery project.

## Materials and methods

Isolation and characterization of peroxidase P7-like gene and Rab-GDI like gene from potential medicinal plants.

### Plant sample collection

Leaf samples of *A. lebbeck* and *M. oleifera* were procured, identified, and confirmed from the Department of Botany, The Women University Multan, Pakistan.

### Extraction of genomic DNA

Genomic DNA from leaf samples of two plant species was extracted using Thermo Scientific GeneJet Plant Genomic DNA Purification Mini kit and stored at −20°C for further investigation (Long et al., 2019). The extracted DNA was analyzed by agarose gel electrophoresis on 1% agarose gel in 1X TAE buffer.

### Amplification of defensin genes from total DNA template through PCR

DNA fragments of plants were amplified by PCR using forward primer 5′-CGCTGCCCCGACGCTTAC-3′ and reverse primer 5′-GACGACTTGGTAGTTGCTGT-3′ using an open reading frame of acknowledged plant defensin genes. The PCR profile for gene amplification was set as follows: 95°C for 3 min followed by 35 cycles of denaturation at 95°C for 1 min, annealing at 48°C for 1 min, extension at 72°C for 1 min, and a final extension at 72°C for 7 min. The PCR products were resolved on 1% agarose gel using 1X TAE buffer at 80 V, followed by ethidium bromide staining. PCR products were purified using a Thermo Scientific GeneJET Gel Extraction Kit. Purified PCR products were sequenced at the DNA sequencing facility of the Center for Applied Molecular Biology, Lahore, Pakistan, under the Access to the Scientific Instrumentation Program of the Higher Education Commission of Pakistan.

### *In silico* analysis

All sequencing results were compiled using DNA Dragon software^1^ (Hepperle, 2011). The consensus sequences obtained from DNA Dragon were checked for their homology with reported sequences presenting nucleotide database using BLASTn and BLASTx programs of NCBI BLAST^2^ (Ingti et al., 2017). The sequences showing significant similarity with query sequences were retrieved and aligned using online Multiple Sequence Alignment tool CLUSTALW^3^ (Thompson et al., 1994). Phylogenetic diversity was analyzed using the neighbor-joining method (Kumar et al., 2016) in the Molecular Evolutionary Genetics Analysis (MEGA) Program, version X (Newman et al., 2016). The predicted amino acid sequence of plant defensin genes was subjected to the ProtParam tool^4^ on the ExPASy server to compute the physicochemical properties (Lim and Yoon, 2018).

The three-dimensional structures of defensin proteins were predicted using I-TASSER, SWISS-MODEL and RaptorX web tools (Fahim et al., 2018). The rough models obtained were subjected to GROMOS96 43B1 executed using Swiss-pdbViewer version 4.0.1 for energy minimization (Amir et al., 2019). PROCHECK and ProSA (Protein Structure Analysis) were used to assess the reliability of the homology models (Kumar et al., 2018). Possible docking sites and ligands were predicted using RaptorX (Wu et al., 2018). CD search and pfam analysis tools were used for the detection of structural and functional domains in plant defensins (Marchler-Bauer et al., 2017; Mistry et al., 2021).

### Statistical analysis

The data were analyzed by simple regression rational analysis (Douwes et al., 2008).

## Results

### DNA purification and amplification of defensin genes

**Figure 1:**
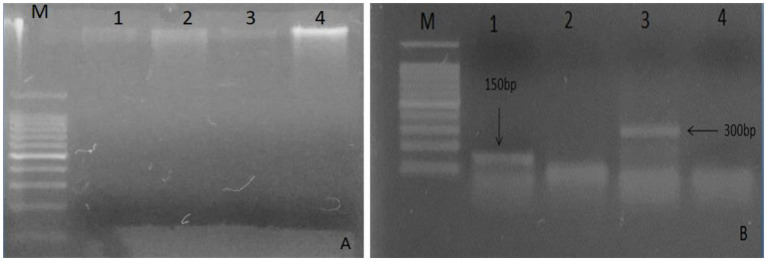
Analysis of total DNA extracted on 1% agarose from plant leaves. **(A)** Lane M: marker 100 bp, lanes 1 and 3 show DNA bands of *Moringa oleifera*, and lanes 2 and 4 show bands of *Albizia lebbeck*; **(B)** Lane M indicates 100 bp DNA ladder, lane 1 shows 150 bp gene of *Moringa oleifera*, and lane 3 shows 300 bp gene of *Albizia lebbeck*.

A good quality DNA (Figure 1A) from leaf samples of both plants were used for defensen gene amplification by PCR. Amplicons appeared as unique bright bands on the agarose gel (Figure 1B). The gene sequence length was ~150 bp for *Moringa oleifera* and >300 bp for *Albizia lebbeck*, as expected.

### Sequence features and phylogenetic analysis of defensin genes

**Figure 2:**
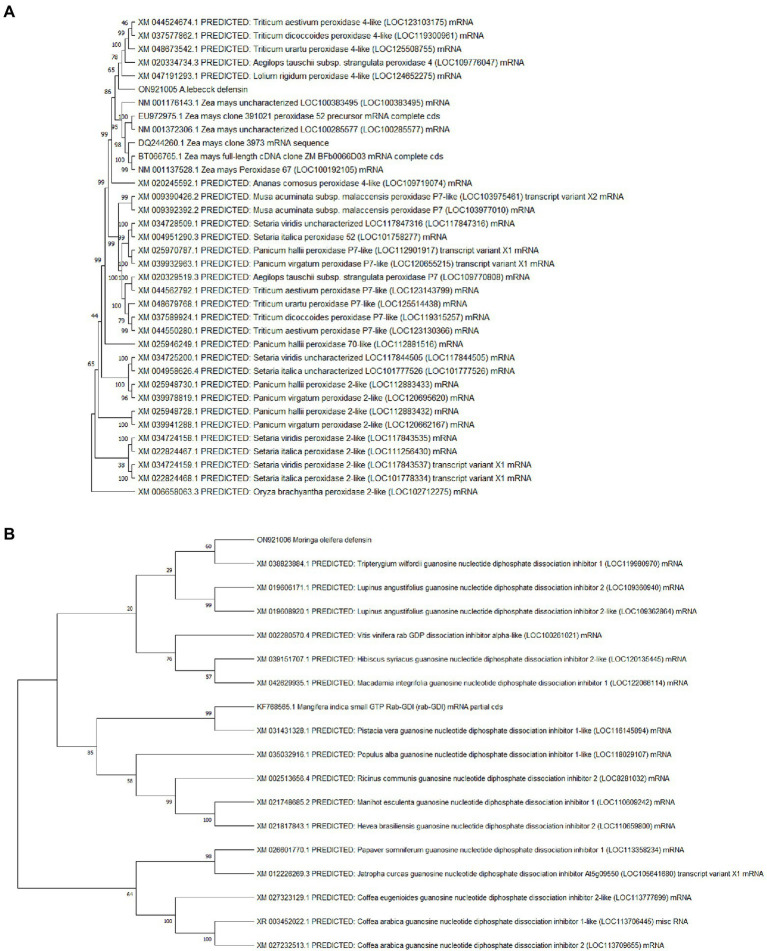
The phylogenetic analysis of **(A)** *A. lebbeck* defensin and **(B)** *M. oleifera* defensin with other plant species by MEGA X from CLUSTAL W alignment. The neighbor-joining method was used to construct the tree with p-distance.

The sequenced fragments of *A. lebbeck* and *M. oleifera* were 300 bp and 317 bp in length containing open reading frames of 204 bp and 174 bp, respectively. Consensus sequences of both fragments were analyzed for their homology with sequences in the NCBI database using BLASTN and BLASTX on the BLAST server.^5^ The *A. lebbeck* defensin gene showed 100% similarity with predicted *Musa acuminata* subsp. *malaccensis* peroxidase P7-like gene (accession number XM_009390426.2) and protein sequence (accession number XP_009388701.1) and the *M. oleifera* defensin gene showed 85% and 91% similarity with predicted *Manihot esculenta* GDP dissociation inhibitor gene sequence (accession number XM_021748685.1) and protein sequence (accession number XP_021604377.1), respectively. The orthologous sequences of *A. lebbeck* and *M. oleifera* defensins were retrieved and subjected to multiple sequence alignment using the online CLUSTALW tool. Phylogenetic analyses retrieved the orthologous polypeptide sequences. Figure 2 shows the phylogenetic trees of *A. lebbeck* and *M. oleifera* defensins. For the defensin-deduced amino acid sequences, 14 published homologous sequences from different plants were used to construct a phylogenetic tree by neighbor-joining distance analysis. For *A. lebbeck*, the topology showed that the polypeptides from four different branches were highly homologous sequences. The *A. lebbeck* polypeptide is clustered with predicted *Musa acuminata* peroxidase P7-like protein, indicating that it is the most recent ancestor of *A. lebbeck* defensin (Figure 2A). The analysis for *M. oleifera* showed clustering of the *M. oleifera* defensin polypeptide with *Manihot esculenta* GDP dissociation inhibitor 1 (Figure 2B). As DNA sequences with more than 75% similarity and protein sequences with more than 25% similarity are considered homologous sequences, we retrieved the sequences of homologous proteins of *A. lebbeck* (*M. acuminata* peroxidase P7-like) and *M. oleifera* defensins (*M. esculenta* GDP dissociation inhibitor 1) to check the properties and structure prediction of novel defensins from understudied medicinal plants.

### Computational analysis of physicochemical properties of *Musa acuminata* peroxidase P7-like and *Manihot esculenta* GDP dissociation inhibitor 1

Table 1 summarizes the ProtParam analysis of physicochemical properties of *M. acuminata* peroxidase P7-like and *M. esculenta* GDP dissociation inhibitor 1. *Musa acuminata* peroxidase P7-like peptide had a predicted molecular weight of ~48 kDa and is neutral with an isoelectric point of 7.02, whereas *M. esculenta* GDP dissociation inhibitor 1 had a molecular weight of ~50 kDa with an acidic isoelectric point of 5.53. Protein low instability and high aliphatic index values predicted that these proteins are heat stable. The grand average of hydropathicity (GRAVY) index indicated that *M. esculenta* GDP dissociation inhibitor 1 is more hydrophilic and has more surface accessibility to interact with water than *M. acuminata* peroxidase P7-like.

**Table 1:** Physicochemical properties of *M. acuminata* peroxidase 7 like and *M. esculenta* GDP dissociation inhibitor 1 by ProtParam.

**S. No.**	**Parameters**	***M. acuminata* peroxidase 7 like**	***M. esculenta* GDP dissociation inhibitor 1**
1	Sequence length	451	444
2	Molecular weight	48,137.46	49,833.88
3	Theoretical isoelectric point	7.02	5.53
4	Instability index	36.39	33.44
5	Aliphatic index	83.41	86.49
6	GRAVY	0.003	−0.254

### Prediction of three-dimensional structure of *Musa acuminata* peroxidase P7-like and *Manihot esculenta* GDP dissociation inhibitor 1

The I-TASSER server with diverse threading templates was used for comparative homology modeling of *M. acuminata* peroxidase P7-like and *M. esculenta* GDP dissociation inhibitor 1. In I-TASSER, the C-score is used to evaluate the result from the consensus of top structural suits. The template modeling score (TM score) is used to measure the homology between structures of templates and models and determine the sequence identity in the structurally aligned area.

**Figure 3:**
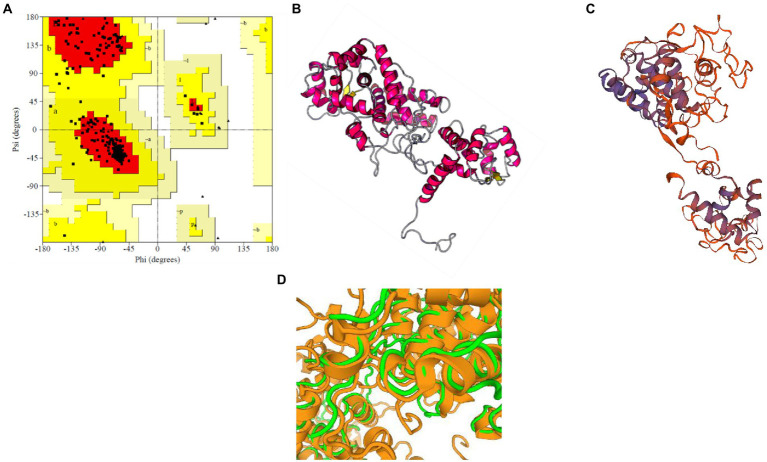
Prediction and validation of 3D structure of *M. acuminata* peroxidase P7-like protein. **(A)** Shows the Ramachandran plot and % residues in the core allowed and disallowed regions. **(B,C)** Show the final modeled structure of *M. acuminata* peroxidase P7-like predicted using I-Tasser and SWISS-MODEL, respectively. **(D)** The 3D structure superposition of template structure and predicted model is shown.

For *M. acuminata* peroxidase P7-like protein, the predicted TM score of the selected model was 0.49 ± 0.15, and RMSD (root mean square deviation) score was 11.6 ± 4.5 Å, observed in the correct configuration. Stereochemical properties were stabilized by energy minimization using Swiss-pdbViewer.^6^ Thepredicted homology model was authenticated using the ProSA and PROCHECK server. Figure 3A shows the Ramachandran plot in which% residues in the core allowed and disallowed regions tells about the acceptability of predicted structure. The maximum likelihood of finding protein residues (>90%) in the core/allowed regions indicatesthat the model built by I-TASSER was the good predicted structure. Figures 3B,C show the final modeled structure of *M. acuminata* peroxidase P7-like. Superimposition of predicted structure with template structure is represented in Figure 3D. PROCHECK and SWISS-MODEL revealed that all residues are in the core/allowed region, with none in the disallowed region, indicating the acceptability of Ramachandran plots (Table 2).

**Table 2:** Ramachandran plot statistics for *M. acuminata* peroxidase 7-like.

	**No. residues**	**Percentage**
Most favored regions [A, B, L]	232	90.6%
Additional allowed regions [a, b, l, p]	24	9.4%
Generously allowed regions [~a, ~b, ~l, ~p]	0	0%
Disallowed regions [XX]	0	0%
Non-glycine and non-proline residues	256	100%
Glycine residues	22	
Proline residues	12	
Total number of residues	292	

**Figure 4:**
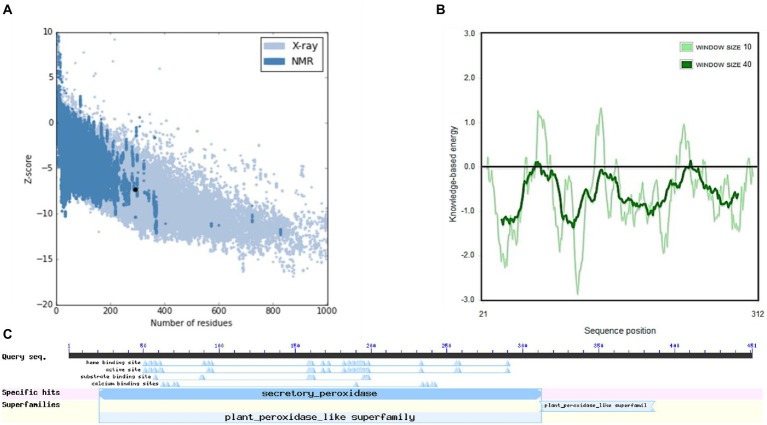
ProSA-web service analysis and domain search of *M. acuminata* peroxidase P7-like protein. **(A)** ProSA-web z-scores of the protein chains present in the Protein Data Bank refer to their length. Light and dark blue colors correspond to X-ray crystallography and NMR spectroscopy, respectively. Large dots highlight the z-scores of *M. acuminata* peroxidase P7-like protein. **(B)** Energy plot of *M. acuminata* peroxidase P7-like protein. **(C)** CD search and Pfam search shows the presence of secretory peroxidase domain in *M. acuminata* peroxidase P7-like protein.

The structure of *M. acuminata* peroxidase P7-like protein was further evaluated using ProSA that mainly considers two predicted structure features: z-score and residue energies plot. Z-score reveals information on the model’s quality, which can be used to measure the amount of total energy of the structure deviating from the energy distribution derived from random conformations. Z-scores outside a range characteristic for native proteins indicate erroneous structures *M. acuminata* peroxidase P7-like had a z-score of −7.29 (Figure 4A), similar to the scores obtained for similar-sized proteins, indicating structure reliability. The residue energy plot depicted the local model quality by plotting energies as a function of amino acid sequence position. The negative values of the *M. acuminata* peroxidase P7-like structure indicates that the predicted model is error-free and acceptable (Figure 4B). To identify the presence of domain in *M. acuminata* protein, pfam and CD search was performed which showed the presence of secretory protein domain (Figure 4C).

**Figure 5:**
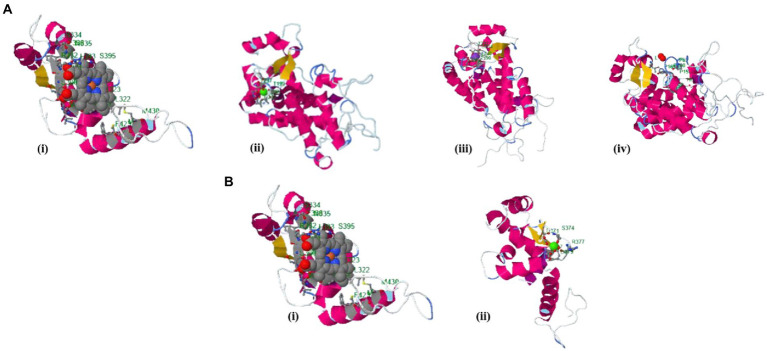
Identified binding sites of *M. acuminata* peroxidase P7-like 3D structure using the raptorX tool. **(A)** Ligand binding pockets identified in domain 1: (I) HEM; (ii) Calcium; (Ca) (iii) Potassium (K); (iv) Pyridine. **(B)** Binding pockets identified in domain 2: (i) HEM; (ii) Calcium (Ca).

The binding site of the targeted protein was predicted using the raptorX tool, with the 3D structure of *M. acuminata* peroxidase 7 shown in Figure 5. The input sequence was processed in two domains, with six binding pockets predicted. Possible ligands that bind with these pockets are HEM, pyridine, Ca, Na, and K. HEM and Ca bind to pockets in both domains, while pyridine, Na, and K bind to pockets in domain 1 only.

**Figure 6:**
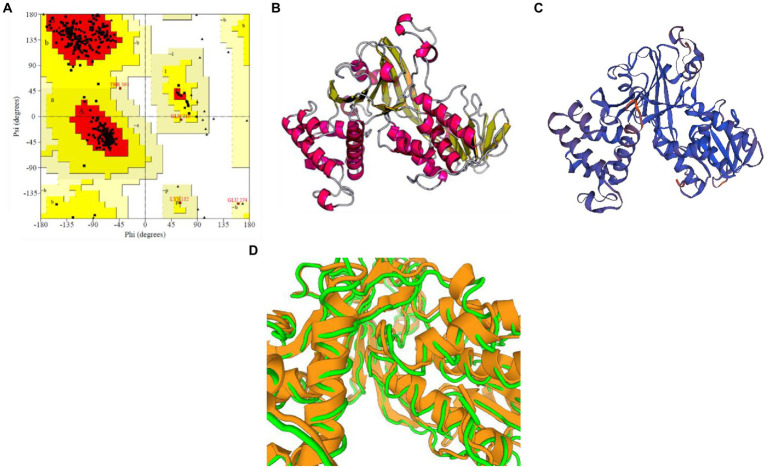
Prediction and validation of 3D structure of *M. esculenta* GDP dissociation inhibitor 1. **(A)** Ramachandran plot of *M. esculenta* GDP dissociation inhibitor 1 protein. The plot calculations for the 3D models were computed in PROCHECK. The most favored regions are in *red*, additional allowed, generously allowed, and disallowed regions are indicated in *yellow*, *light yellow*, and *white*, respectively. **(B,C)** The final 3D structure of *M. esculenta* GDP dissociation inhibitor 1 predicted using I-tasser and SWISS-MODEL, respectively. The α-helix is represented by *red helix*, β-sheet by *yellow arrows*, and loops by *gray lines* **(D)**.

The selected model of *M. esculenta* GDP dissociation inhibitor 1 had a predicted TM score of 0.89 ± 0.07 and RMSD score of 4.4 ± 2.9 Å, observed in the correct configuration. Stereochemical properties were stabilized by energy minimization using Swiss-pdbViewer. The predicted homology model was authenticated using ProSA and PROCHECK. Figure 6A shows the Ramachandran plot and % of the residues in the core allowed and disallowed regions. The maximum likelihood of finding protein residues (>90%) in the core/allowed regions indicates that the predicted model is of good stereochemical quality. PROCHECK revealed that all residues are in the core/allowed region, with none in the disallowed region, indicating the acceptability of Ramachandran plots (Table 3).

**Table 3:** Ramachandran plot statistics for *M. esculenta* GDP dissociation inhibitor 1.

	**No. residues**	**Percentage**
Most favored regions [A, B, L]	365	93.8%
Additional allowed regions [a, b, l, p]	20	5.1%
Generously allowed regions [~a, ~b, ~l, ~p]	4	1%
Disallowed regions [XX]	0	0%
Non-glycine and non-proline residues	389	100%
Glycine residues	31	
Proline residues	22	
Total number of residues	444	

**Figure 7:**
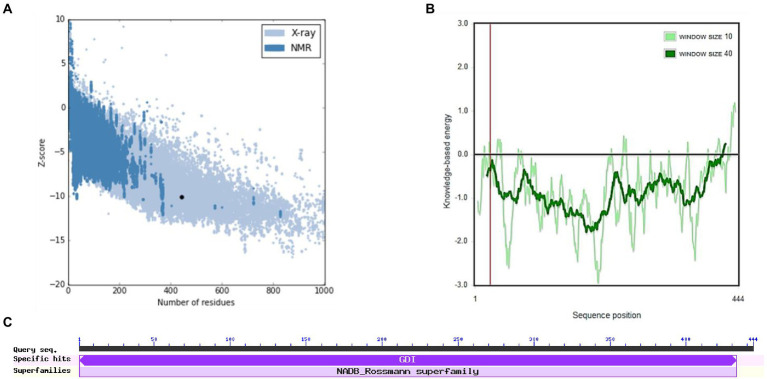
ProSA-web service analysis and domain search of *M. esculenta* GDP dissociation inhibitor 1. **(A)** ProSA-web z-scores of all protein chains present in the Protein Data Bank refer to their length. Light and dark blue colors correspond to X-ray crystallography and NMR spectroscopy, respectively. Large dots highlight the z-scores of *M. esculenta* GDP dissociation inhibitor 1. **(B)** Energy plot of *M. esculenta* GDP dissociation inhibitor 1. **(C)** CD search and Pfam search shows the presence of GDI domain in *M. esculenta* GDP dissociation inhibitor 1.

Figures 6B,C show the final modeled structure of *M. esculenta* GDP dissociation inhibitor 1, which was further evaluated using ProSA. Superimposition of predicted structure with template structure is represented in Figure 6D. *Manihot esculenta* GDP dissociation inhibitor 1 had a z-score of −10.11 (Figure 7A), similar to the scores of similar-sized proteins, indicating structure reliability. The residue energy plot depicted local model quality by plotting energies as a function of amino acid sequence position. The negative values of the residue energy plot of *M. esculenta* GDP dissociation inhibitor 1 structure indicate that predicted model is error-free and acceptable (Figure 7B). To identify the presence of domain in *M. esculenta* GDP dissociation inhibitor, pfam and CD search was performed which showed the presence of GDI domain (Figure 7C).

**Figure 8:**
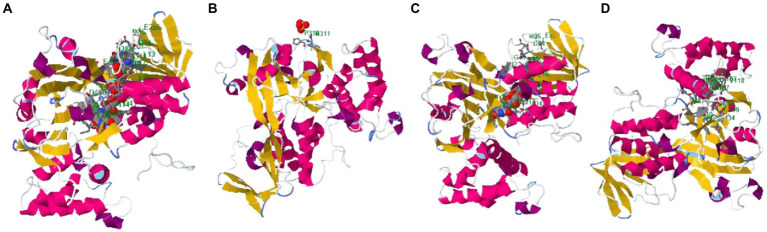
Identified binding sites of *M. esculenta* GDP dissociation inhibitor 1 using the raptorX tool. Four binding pockets were identified: **(A)** Dihydroflavine-adenine dinucleotide (FDA), **(B)** sulfate ion (SO_4_^2−^), **(C)** chloride ion (Cl^−^), and **(D)** galactose-uridine-5′-diphosphate (GDU).

The binding site of the targeted protein was predicted using the raptorX tool, with the 3D structure of *M. esculenta* GDP dissociation inhibitor 1 shown in Figure 8. The input sequence was processed, with four binding pockets predicted. Possible ligands that bind with these pockets are dihydroflavine-adenine dinucleotide (FDA), sulfate ion (SO_4_^2−^), chloride ion (Cl^−^), and galactose-uridine-5′-diphosphate (GDU).

## Discussion

Plants have the ability to integrate divergent signaling pathways to allow appropriate defense responses against various stresses (Brenner et al., 2012; Overmyer et al., 2018) Therefore, overexpression of a single gene can have a negative (i.e., susceptibility) or positive effect (i.e., tolerance) on the plant response to other stresses. These genes also serve as a promising source for engineering disease resistance traits in plants, enhancing self-defense mechanisms and reducing the requirement for supplementary chemical microbicides (Boukhatem et al., 2014). In this study, *in vitro* and *in silico* methods were used to isolate and characterize novel plant defending family-specific antimicrobial genes from *Albizia lebecck* and *Moringa oleifera*. Same kind of study on defensin gene isolation from *Saccharum officinarum* was done by De-Paula and colleagues, who analyzed six sugar cane putative defensins (De-Paula et al., 2008). In our study, primers were designed on a consensus sequence of 3–5 aligned sequences retrieved from the NCBI database. Highly similar sequences that exhibited 75%–100% similarity with the primary sequence were selected for alignment through the BLAST tool. In a recent study on one class of defensin genes—biodefensin—Rawat et al. (2017) isolated the full length of this gene encoding about 80 amino acids from *Brassica juncea* (family Brassicaceae). The authors reported that the gene had high levels of similarity with gamma thionin and knottin families of plant AMPs. Our isolates also showed that the biodefensin had similarities with defense genes in other plants. Studies have shown that gene transcription is significantly enhanced under stress, confirming the inducible defense response of AMP in plants (Rawat et al., 2017). The plant defensins Rs-AFP1 and Rs-AFP2 from radish (*Raphanus sativus*) is an example of potent antifungal proteins. The exact process involved in the antifungal function of these genes needs further study, but they likely target the cell membranes of microbes. These genes may also initiate other mechanisms involved in plant defense (Souza et al., 2017).

We used many online bioinformatics tools to study the physicochemical and functional properties, and also structural analyses of our novel defensin genes. Same kind of comparative analysis on Kisspeptin Receptors and their physicochemical characterization was done by Mukesh Kumar and his co-workers (Kumar et al., 2018). We predicted the 3-D structures of defensin proteins using I-TASSER and RaptorX web tools. Our novel gene, isolated from *Moringa oleifera* that showing homology with the GDP dissociation inhibitor gene, may function in membrane transport, as found in the GDP dissociation inhibitor (Palme, 1997; Zarsky et al., 1997). The GDP dissociation inhibitor gene regulates nucleotide states and subcellular localization of Rab/Ypt proteins (Ueda et al., 1998). Rab3a-GDI (Rab guanosine nucleotide diphosphate dissociaton inhibitor protein), first isolated from bovine cytosol (Sasaki et al., 1991), inhibits the dissociation of GDP to Rab3a and the binding of GTP to Rab3a, and thus has a potential function in regulating Rab GTPases (Burstein and Macara, 1992) and secretory pathways (Andreeva et al., 1997). While we have not tested the function of our novel gene, but its homology with GDP dissociation inhibitor and function in membrane vesicular transport strongly suggest that it will also inhibit the entry of microbes across the membrane, thus preventing infection. Our other novel gene, isolated from *Albizzia lebecck* showed 100% similarity with predicted *Musa acuminata* subsp. *malaccensis* peroxidase P7-like gene. In a cold tolerance study of *Musa* spp. ‘Dajiao’, He et al. (2018) found that membrane-bound peroxidases potentially decrease lipid peroxidation and play a role in maintaining leaf cell water potential, contributing to plant defense against cold stress. Other researchers also found that membrane peroxidases are involved in the H_2_O_2_ scavenging system and ROS signaling networks (Passardi et al., 2004). The role of peroxidases in plant defense is elaborated by their activity on cell walls through the oxidation of phenolic compounds by reducing H_2_O_2_ under normal and stressed conditions (Almagro et al., 2009). Another study identified three defense enzymes—phenylalanine ammonia-lyase (PAL), peroxidase (PO), and polyphenol oxidase (PPO)—involved in phenol synthesis and oxidation in banana roots infected with *Radopholussimilis*, which strengthened cell walls. Wounded roots had significantly higher PO levels than control roots, they also reported enhanced PO activity in banana roots inoculated with fungus within 24 h of infection (Wuyts et al., 2006). These results and the homology of our isolated gene with *Musa acuminata* subsp. *malaccensis* peroxidase P7-like gene strongly suggests that Albizzin has the same functional potential in the defense mechanism of plants and can serve its function against fungal diseases.

## Conclusion

No doubt huge efforts are being made on genetic researches all over the world to discover more potent medicinal plants with beneficial gene products. However, still a large number of medicinal plants are at the need to be investigated for their possible defensive values. This study suggests a strategy for isolation of effective candidate genes that are part of signal transduction and defense system of plants. Examining the efficiency of these genes in practice is key to understand its role in defense mechanism. Screening of defensin gene from *M. oliefera* and *A*. *lebecck* resulted in isolation of GDP dissociation inhibitor like gene and peroxidase P7-like gene, respectively. These genes are very important as their role in membrane transport, regulation of nucleotide states, subcellular localization, free radical sacavenging properties and signaling of ROS. Further investigation on physical characterization and interaction of gene with downstream stress-responsive and growth and developmental-related genes of plant breeds with these genes would be worth examining whether these plants have enhanced resistance or tolerance to multiple biotic and abiotic stresses. Hence these genes would be potential candidate for breeding of plants with high resistance and strong defense. Structural characterization and biological interactions of GDP dissociation inhibitor and peroxidase P7-like gene from *M. oliefera* and *A*. *lebecck* respectively, revealed in this study conclude that these genes can serve for the synthesis of transgenic plants that will be more stress resistant and can give better crop yield in the future.

## Data availability statement

The original contributions presented in the study are publicly available. This data can be found at: ON921005, ON921006.

## Author contributions

Research work was supervised by RJ and AI. Material preparation, data collection, and analysis were performed by UH, RJ, and FD. The manuscript was finalized by HN and GM. Article review, editing, and proof reading was done by FZ, MN, AZ, and KS. All authors contributed to the article and approved the submitted version.

## Funding

The research leading to these results received funding from Higher Education Commission, Government of Pakistan under grant agreement no. 21-1603/SRGP/R&D/HEC/2017.

## Conflict of interest

The authors declare that the research was conducted in the absence of any commercial or financial relationships that could be construed as a potential conflict of interest.

## Publisher’s note

All claims expressed in this article are solely those of the authors and do not necessarily represent those of their affiliated organizations, or those of the publisher, the editors and the reviewers. Any product that may be evaluated in this article, or claim that may be made by its manufacturer, is not guaranteed or endorsed by the publisher.

## Footnotes

^1^ http://www.dna-dragon.com/

^2^ http://blast.ncbi.nlm.nih.gov/Blast.cgi

^3^ http://www.genome.jp/tools/clustalw/

^4^ https://web.expasy.org/protparam/

^5^ https://blast.ncbi.nlm.nih.gov/Blast.cgi

^6^ https://spdbv.unil.ch/
